# Case Report: Simultaneous covered stenting for aortic coarctation and significant PDA in low and very low birth weight preterm infants: technical insights from two cases

**DOI:** 10.3389/fcvm.2025.1611156

**Published:** 2025-09-04

**Authors:** Nathalie Mini, Ehrenfried Schindler, Katinka Breuer, Marian Mikus, Martin B. E. Schneider

**Affiliations:** ^1^ Cardiac Catheterization Laboratories, German Paediatric Heart Center, University Hospital Bonn, Bonn, Germany; ^2^ Department of Anesthesiology and Intensive Care Medicine, University Hospital Bonn, Bonn, Germany; ^3^ Department of Neonatologie and Paediatric Intensive Care, Altona Children’s Hospital, Hamburg, Germany

**Keywords:** severe coarctation of the aorta, prematurity, covered coronary stent, CoA in prematurity, CaA stenting

## Abstract

**Background:**

To the best of our knowledge, no study to date has reported the use of covered stents in preterm and low weight (LW) preterm babies for the combined treatment of aortic coarctation (CoA) and persistent patent ductus arteriosus (PDA) in these critically ill patients.

**Patients and methods:**

In 2023, two premature infants with isolated critical CoA and a window-shaped PDA were treated with Biotronik PK Papyrus covered coronary stents. The indications for intervention in the first patient included a significant, short PDA with respiratory deterioration and necrotizing enterocolitis (NEC), despite prior CoA treatment using a premounted coronary stent and ongoing ibuprofen therapy, which failed to close the PDA. The second patient presented with impaired left-ventricular (LV) function, renal insufficiency, and NEC. Echocardiography was used both to assess the outcome of the intervention and for follow-up. The patency of the PDA was documented at 12 and 24 h postintervention.

**Results:**

Patient 1, with a gestational age (GA) of 32 weeks and a birth weight of 1,400 g, and Patient 2, with a GA of 30 weeks and a birth weight of 1,900 g, underwent the procedure at 20 and 11 days of age, respectively. Both patients successfully received a PK Papyrus covered stent implantation. Angiographic imaging immediately following the intervention revealed evidence of a residual shunt in each patient. However, at 12 h postprocedure, no residual PDA shunt was detected on echocardiography. Both patients underwent successful surgical repair 7 and 10 months later, with complete removal of the 15 mm stents.

**Conclusion:**

In our rare and small cohort, covered stenting for CoA and PDA proved effective and safe in low-weight and very-low-weight premature infants, improving coarctation, achieving complete PDA closure, and offering the potential for full stent removal. The small sample size reflects the cohort's critical specificity, and the findings require validation in larger populations.

## Introduction

Early repair of aortic coarctation (CoA) in newborns is typically feasible and associated with positive outcomes (1). However, some infants with CoA are not suitable candidates for neonatal correction due to factors such as impaired left-ventricular (LV) function, extremely low birth weight, prematurity, and intraventricular haemorrhage. In these cases, prostaglandin E1 remains the preferred treatment to maintain ductal patency, serving as a bridge to eventual surgical intervention. Previous studies (2) have demonstrated that stenting of CoA in very-low- (VLW) and extremely low-birth-weight preterm babies using noncovered coronary stents is a feasible and effective approach. This strategy serves as a bridge to surgical repair while maintaining a minimal gradient, particularly in very ill preterm patients with renal insufficiency and those who have experienced side effects from prostaglandin E1. When performed under echocardiography guidance, the procedure is straightforward and eliminates the need for contrast agents, which is particularly beneficial for premature infants with renal insufficiency, reducing the risk of renal complications (3).

The incidence of patent ductus arteriosus (PDA) in preterm infants ≤ 28 weeks' gestational age (GA) exceeds 50%. Although spontaneous closure rates are high, historical practices have led to medical or surgical therapy in 60%–70% of preterm infants < 28 weeks (4). The treatment of CoA and nonclosed PDA in LW and VLW infants presents particular challenges, especially when the PDA is short and wide.

This report aims to review the results of our centre in the simultaneous treatment of CoA stenting and window-shaped PDA using a covered stent in two LW infants.

## Patients and methods

In 2023, two LW and VLW preterm infants with isolated CoA and window-shaped PDA underwent simultaneous treatment with covered stents to address both conditions. The primary indications for this emergency intervention were to relieve severely impaired LV function and improve renal perfusion because both infants were experiencing renal failure.

The procedure was performed via the left carotid artery in the Patient 1 and the femoral artery in Patient 2, with local anaesthesia (MecainR 10 mg/ml, 1 ml/kg) administered before and after the intervention. Ultrasound guidance was used for all punctures. A 24-gauge needle was employed to access the femoral artery, followed by a 0.14 guidewire (steerable guidewire Wizdom™) to introduce a short sheath. The coronary stent was then used to stent the aortic coarctation, with premounted covered coronary stents (Biotronik PK Papyrus covered coronary stents) used in the two patients.

Postprocedure echocardiography was performed at 24, 48, and 72 h to evaluate the outcome of the intervention. Key factors, including improvement in LV function, the need for reintervention, recurrence of restenosis, and the timing and results of subsequent surgical repair, were meticulously documented.

## Description of patients

### Patient 1

A premature infant with a GA of 32 weeks and a birth weight of 1,200 g presented with isolated critical CoA and a large, open PDA, managed with prostaglandin E1. The patient was also diagnosed with congenital chylothorax and secondary pleural effusion. The indication for CoA stenting included severely impaired LV function, necrotizing enterocolitis (NEC), and renal insufficiency. When the patient was 17 days old, the aortic coarctation was treated with the implantation of a noncovered coronary stent (4.5X 9 mm). Despite treatment with ibuprofen, the large, window-shaped PDA failed to close, and the patient's condition deteriorated, with severe pulmonary overcirculation and low cardiac output. Surgical ligation of the PDA was considered high risk due to potential stent compression during surgery. Additionally, the short, wide PDA was unsuitable for closure with an occluder device because of the presence of the CoA stent.

### Patient 2

A premature infant with a GA of 30 weeks and a birth weight of 1,450 g presented with severe isolated CoA with a large and short PDA. The patient was diagnosed with VACTERL association, including oesophageal atresia with tracheoesophageal fistula Type 3b, requiring a tracheostomy. The primary indication for CoA stenting was severely impaired LV function.

### Ethical statement

According to the decision of the local ethical committee (Running Number 2025-112-BO), informed consent and patient agreement were waived due to the retrospective design of the study.

## Results

At the time of the intervention, Patient 1 weighed 1,400 g and was 20 days old while Patient 2 weighed 1,900 g and was 11 days old. In Patient 1, the left carotid artery was accessed due to spasm in the femoral arteries during puncture. In contrast, the femoral artery was successfully used for Patient 2. A 3.3-French short sheath was utilized in Patient 1 and a 4-French sheath in Patient 2. The procedure was performed under fluoroscopy.

**Figure 1:**
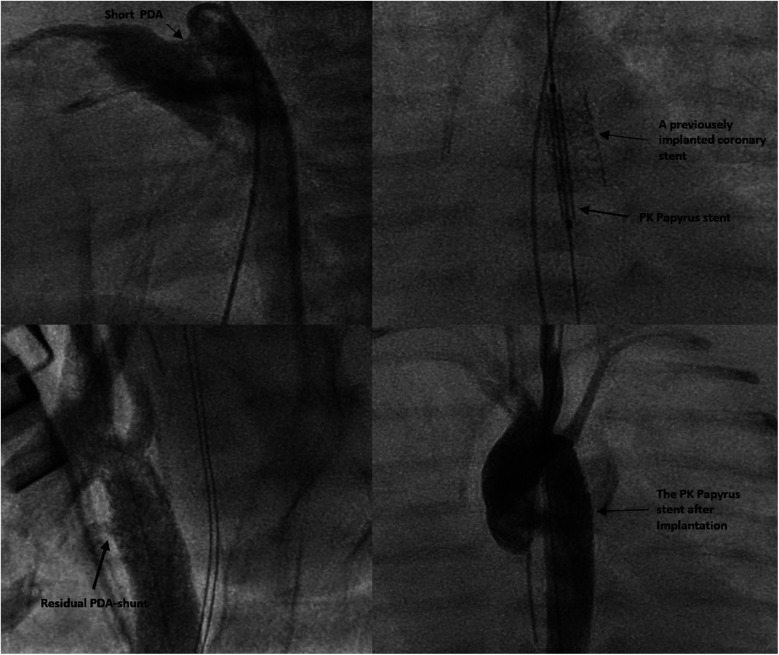
Demonstration of the intervention details in patient 1.

**Figure 2:**
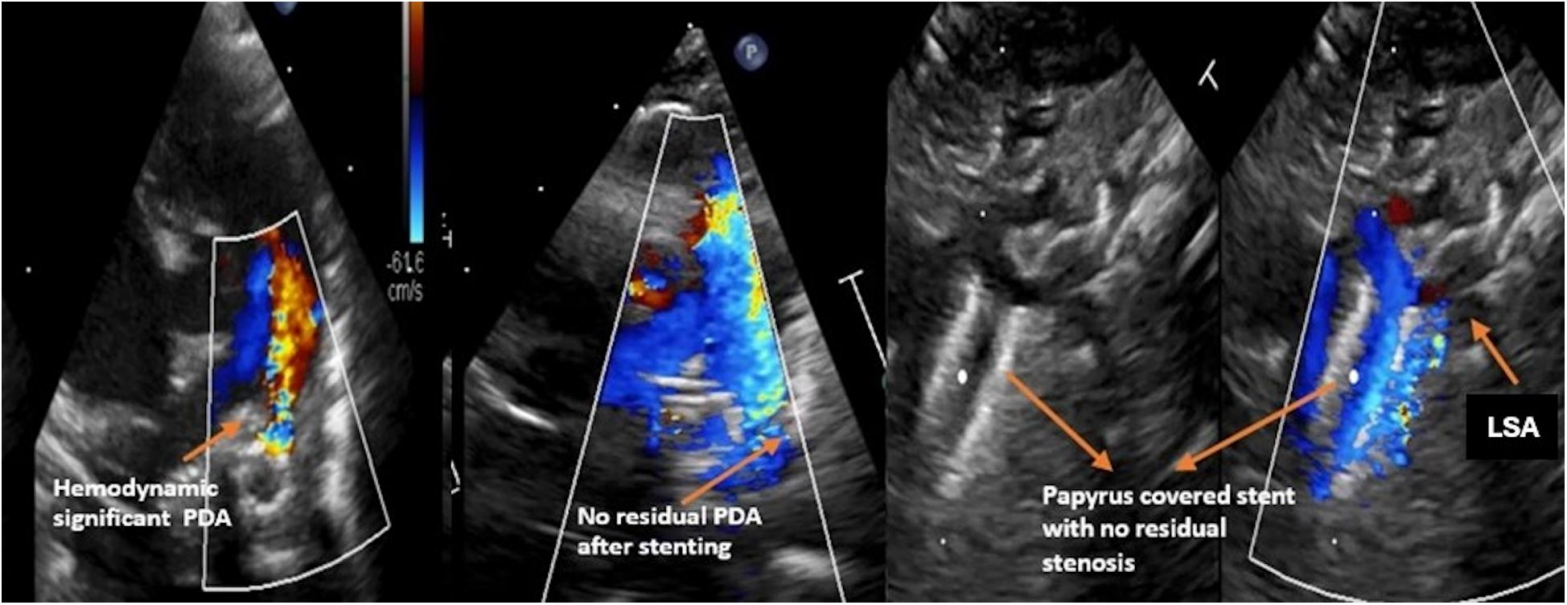
Demonstration of the PDA shunt before and after the intervention, as well as the position of the stent in patient 1.

In Patient 1, who already had a stent in place for the CoA, a PK Papyrus covered coronary stent (5 × 15 mm) was inserted into the existing stent, and angiography confirmed complete coverage of the PDA (Figures 1, 2).

For Patient 2, a planned surgery for oesophageal atresia was scheduled for the following day, alongside a simultaneous interventional PDA closure and CoA stenting. After the CoA was stented with a premounted 4 × 9 mm coronary stent, the PDA was visualized angiographically and echocardiographically, with the stent protruding into the large and short PDA. Consequently, the decision was made to implant an additional covered stent to exclude the large PDA and stabilize the initial stent.

**Figure 3:**
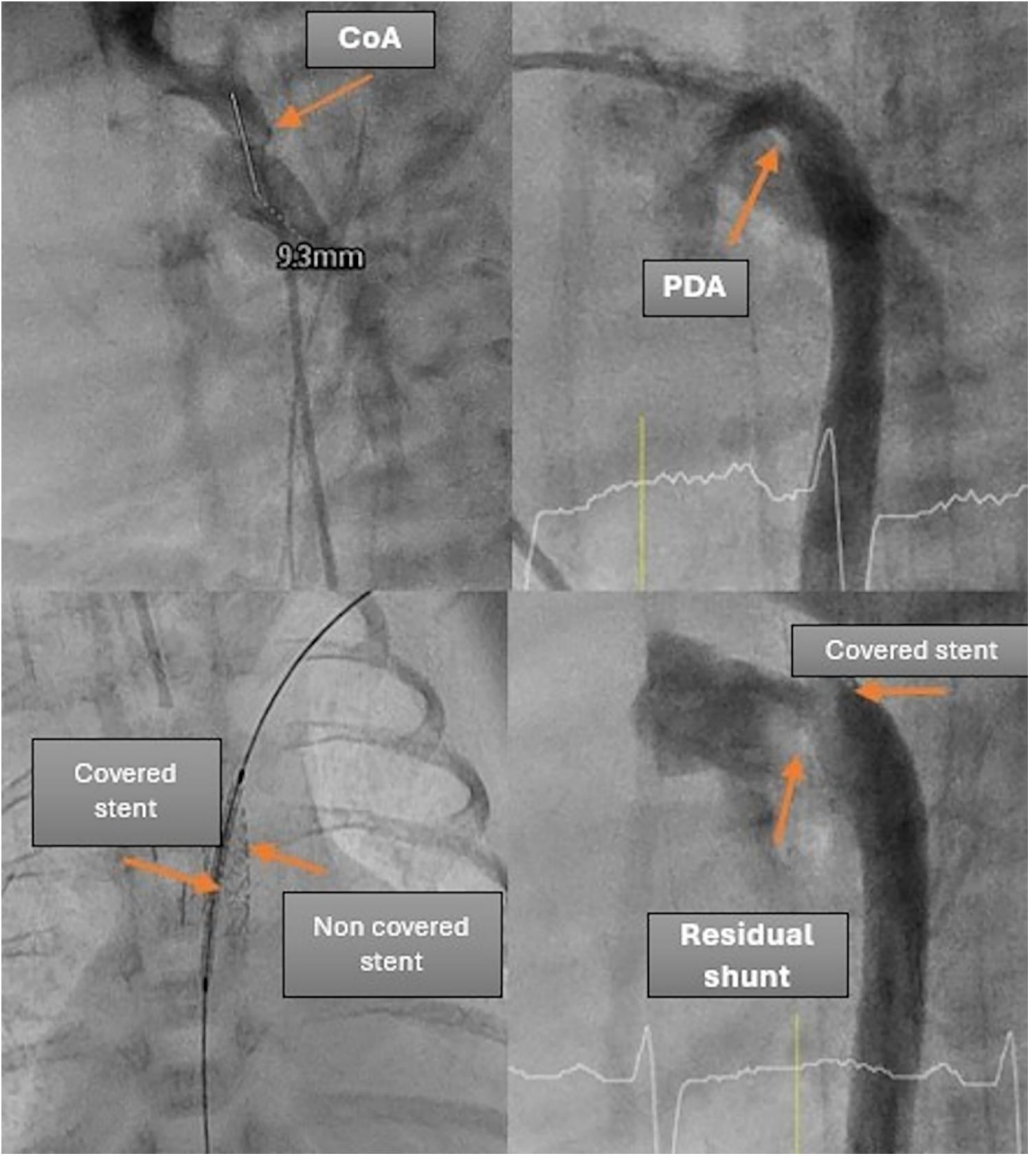
Demonstration of the intervention details in patient 2.

**Figure 4:**
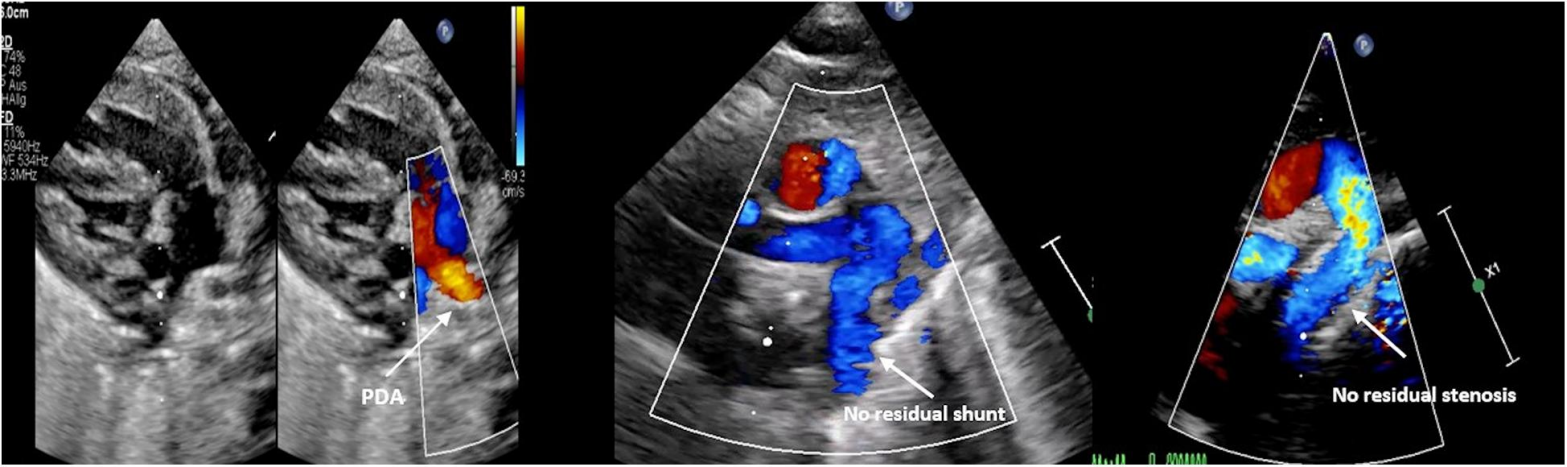
Demonstration of the echocardiographic findings before and after the intervention in patient 2.

A 4 × 15 mm PK Papyrus covered stent was inserted through the initial stent and implanted, completely covering the PDA. In both patients, the procedure was complication free. The patency of the left subclavian artery (LSA) was confirmed through angiography. Immediately after the intervention, angiography (Figure 3) revealed a residual shunt in the PDA despite the covered stent; however, at 12 h postintervention, no residual shunt was observed (Figure 4). The femoral artery spasm in Patient 1 was relieved 24 h after the intervention, with no signs of stenosis or thrombosis.

Following the intervention, both patients showed significant improvement in LV function and a dramatic enhancement in their respiratory status. Patient 1 was successfully extubated for the first time after the procedure while Patient 2 remained with a tracheostomy due to noncardiac related issues. In the echocardiographic follow-up, the LV function improved on the first day of intervention. Renal parameters gradually normalized, and the respiratory status also improved in Patient 1, who was successfully extubated. Patient 2 had a tracheostomy and had undergone multiple surgeries due to oesophageal atresia. Surgical repair of the CoA and removal of the stent were performed in Patient 1 at 10 months of age and a weight of 5 kg and in Patient 2 at 7 months of age and a weight of 6 kg. In both cases, the stent, despite its 15 mm length, was successfully and completely removed.

## Discussion

Stenting for critical CoA in LW and VLW, critically ill infants using premounted coronary stents has proven to be both safe and effective This approach serves as a bridge to surgical repair, alleviating CoA-related renal insufficiency and NEC while also reducing the side effects associated with high doses of prostaglandin E1 (2, 3). Following stenting, the PDA typically closes either spontaneously or through medical therapy with ibuprofen. However, in some cases, large, hemodynamically significant PDAs persist despite medical treatment. These residual PDAs can lead to pulmonary overcirculation, worsening respiratory status, and decreased cardiac output, ultimately prolonging the duration of intubation and extending the intensive care unit (ICU) stay.

Simultaneous interventional treatment of critical CoA and PDA, or the interventional closure of PDA after stenting in preterm infants, presents significant challenges due to the close anatomical relationship between the PDA and the CoA. Although the transcatheter closure of isolated PDA using various devices can be difficult and carries the risk of complications such as aortic obstruction or left pulmonary artery stenosis in preterm and VLBW infants (5, 6), the procedure becomes even more complex when the PDA is associated with CoA or a stent in the CoA.

Combined transcatheter treatment of CoA and PDA has been documented in children over 6 years of age and in adults (7, 8). However, no reports have yet described the use of this approach in preterm or low-birth-weight (LBW) infants (<2 kg).

In this study, we reviewed our experience with the use of covered coronary stents in critically ill preterm infants with LBW or VLBW who present with severe CoA and a large, short, window-shaped PDA.

For these cases, we utilized the Biotronik PK Papyrus covered coronary stent to simultaneously treat CoA and exclude the PDA. These premounted covered stents can be delivered through a 3.3-French sheath, making them highly suitable for VLBW and extremely LBW infants.

The PK Papyrus stents are available in various sizes, ranging from 2.5–5 mm, with expandable diameters of approximately 2.83–4.63 mm (Figure), making them well suited for the small aortic dimensions in LW and VLW babies.

The stents in our patients were successfully implanted without complications and proved to be both effective and adequate for treating CoA, resulting in a significant reduction of pressure gradients and successful exclusion of the PDA shunts.

Immediately after implantation, angiography revealed a residual shunt in each patient's PDA. However, echocardiographic evaluation 12 h postintervention showed no evidence of a residual shunt. This observation is consistent with studies conducted on patients over 6 years old and in adult patients who underwent similar interventions (4).

Despite the flexible stent sizes and their compatibility with insertion through a 3.3-French sheath, the available 15-mm length may not be ideal for small infants. Additionally, surgical stent removal can be challenging for the surgeon. However, in our patients, the stents were successfully and completely removed.

To better accommodate the needs of preterm babies, our catheter lab has recently acquired Bentley BeGraft coronary stents. These stents range in diameter from 2.5–5 mm and are available in shorter lengths of 8 and 12 mm, making them a promising option for this patient population. However, compared to the PK Papyrus stent, the BeGraft coronary stent is more compatible with a 4-French sheath rather than a 3-French sheath.

It is important to note that the use of covered stents in very small patients may lead to obstruction of the left subclavian artery if the stent is inserted without a long sheath. The sheath helps ensure proper placement of the stent and allows for angiographic confirmation of its position relative to the subclavian artery. Therefore, it is crucial to carefully assess the stent's position using echocardiography to ensure that the entrance to the left subclavian artery remains clear, thus preventing potential complications.

## Conclusion

The simultaneous transcatheter treatment of CoA and PDA using a covered coronary stent in our rare and small cohort with two critically ill, LW and VLW preterm infants was both safe and feasible. This approach may be the optimal choice to minimize the need for reintervention, shorten procedural duration, and reduce the potential risk of PDA device-induced pulmonary obstruction, which may be exacerbated by the presence of a CoA stent in these patients. The limited number of patients in this report reflects the highly specific and critical nature of the cohort, and the results should be validated in larger patient populations.

## Data Availability

The original contributions presented in the study are included in the article/Supplementary Material, further inquiries can be directed to the corresponding author.
